# Motivations for Enrolment and Dropout of First-Year Undergraduate Nursing Students: A Pilot Multimethod Study

**DOI:** 10.3390/nursrep14040254

**Published:** 2024-11-13

**Authors:** Elena Viottini, Alice Ferrero, Beatrice Albanesi, Johnny Acquaro, Giampiera Bulfone, Francesca Condemi, Donatella D’Accolti, Azzurra Massimi, Elisa Mattiussi, Roberta Sturaro, Alessio Conti, Valerio Dimonte

**Affiliations:** 1 Department of Biomedicine and Prevention, University of Rome “Tor Vergata”, 00133 Rome, Italy; elena.viottini@unito.it; 2 Department of Public Health and Pediatrics, University of Torino, 10126 Torino, Italy; alice.ferrero336@edu.unito.it (A.F.); beatrice.albanesi@unito.it (B.A.); valerio.dimonte@unito.it (V.D.); 3 Bachelor of Science in Nursing Programme, Department of Public Health and Pediatrics, University of Torino, 10126 Torino, Italy; johnny.acquaro@unito.it (J.A.); francesca.condemi@unito.it (F.C.); roberta.sturaro@unito.it (R.S.); 4 Department of Medical, Surgical Science and Advanced Technology “G. Ingrassia”, University of Catania, 95123 Catania, Italy; giampiera.bulfone@unict.it; 5 Department of Precision and Regenerative Medicine and Ionian Area—(DiMePRe-J), “Aldo Moro” University of Bari, 70121 Bari, Italy; donatella.daccolti@uniba.it; 6 Department of Public Health and Infectious Diseases, Sapienza University of Rome, 00185 Rome, Italy; azzurra.massimi@uniroma1.it; 7 Dipartimento di Assistenza Territoriale, Azienda Sanitaria Universitaria Friuli Centrale, 33100 Udine, Italy; mattiussielisa@gmail.com; 8 Department of Clinical and Biological Sciences, University of Torino, 10043 Torino, Italy; 9 Direction of Health Professions, Azienda Ospedaliero-Universitaria Città della Salute e della Scienza, 10126 Torino, Italy

**Keywords:** undergraduate nursing students, enrolment, dropout, first-year, nursing workforce

## Abstract

Background/Objectives: Higher education institutions must improve the attractiveness and retention of the nursing profession to address the widespread shortage. This pilot multimethod study aimed to preliminarily understand the relationship between motivations for enrolment and dropout among first-year undergraduate nursing students. Methods: A two-step approach was conducted among first-year nursing students from five Italian universities involving: (a) a baseline quantitative online survey collecting their characteristics and motivations for enrolment; (b) a follow-up semi-structured interview qualitative data collection among students who dropped out. Descriptive and inferential statistics were used to describe the motivations for enrolment and differences between universities. Dropout motivations emerged from inductive content analysis, with data categorisation according to Urwin’s framework. Results: A total of 759 students completed the online survey. Primary motivations for enrolment included the desire to be useful (88.8%), help suffering people (84.3%), and find employment (74.2%); 22.3% cited unsuccessful admission to another university as motivation for enrolment. Of the 141 students who discontinued, 31 were interviewed (22%). Eleven categories and three themes were identified. More than half of the participants dropped out due to interest in other courses and lack of aptitude, while a smaller number cited personal circumstances. Other motivations for dropout were related to negative learning environments or feelings and difficulties related to course characteristics. Conclusions: This study provides an initial insight into these complex phenomena that will be instrumental in understanding data from an Italian multicenter cohort study. The findings can inform recommendations and strategies to strengthen the future nursing workforce.

## 1. Introduction

Nurses have been defined as the lifeblood of healthcare systems, as they alone comprise nearly 50% of the global healthcare workforce, with a pivotal role in achieving universal health coverage [1]. This is because they are responsible for providing quality healthcare services that improve health outcomes for people and communities. However, the current shortage of nurses is becoming increasingly widespread throughout the world [2].

The World Health Organization (WHO) has estimated an additional 9 million nurses’ requirement by 2030 [3], with at least 60,000 more needed only in Italy [4]. This condition is the result of various factors, including inadequate investment in nursing staff, a lack of policies that promote the recruitment and retention of nurses, as well as inappropriate salaries, unattractive working conditions, high turnover, and negative cultural perceptions of the profession [4,5]. It is imperative that all institutions take action and focus on developing effective recruitment and retention strategies to reduce the shortage of nurses [6]. This issue cannot be solved unless higher education institutions invest in educating the next generation of nurses and graduate more students who aspire to become registered nurses [2].

In Italy, becoming a nurse requires completing a three-year Bachelor of Science in Nursing (BSc Nursing). Admission is limited, with the number of places available determined annually by law, requiring all candidates to pass an entrance exam. After the selection process, a list of eligible students for enrolment is published [7]. Despite the increase in the number of places available for Bachelor of Science in Nursing (BSc Nursing) programmes in Italian universities in recent years, applications have been in continuous decline from −8.6% in 2023 to −10.1% in 2024 [8].

Various motivations have been identified for individuals to enrol in the BSc Nursing programme. These motivations can be intrinsic, referring to doing something with the willingness to increase the sense of personal fulfilment and satisfaction rather than for its consequences, for example, altruistic drive or personal interest; extrinsic, regulated by external influences or perceived rewards, such as influences from family members or friends, a job or career opportunities; previous experiences in healthcare context including voluntary activities, illness condition or death of loved ones [9]. Additionally, students who are unsure about their path or choose nursing because they cannot attend other university courses may be considered unmotivated [10]. Concurrently, the workforce shortage is further exacerbated by the dropout phenomenon within nursing programmes, where students leave before obtaining their degrees. According to Urwin’s framework developed in 2010, factors contributing to dropout may be individual, such as socio-demographic characteristics, academic abilities or family and working commitment (micro-level); institutional factors, such as programme curricula and organisation (meso-level); and political aspects and career prospects within the nursing profession (macro-level) [11].

Although several studies [12,13,14,15,16,17,18,19,20,21,22,23,24,25,26,27,28,29,30,31,32,33,34,35,36,37,38] have been conducted focusing on the motivations for enrolment and dropout of nursing students, the reduction in the number of applications to the BSc Nursing programmes, combined with the progressive reduction in the inflow of nurses in the workforce due to academic discontinuation, is still calling institutions to understand the reasons underlying these two phenomena. Furthermore, it is implicit that the health and social policies at the national level and the local context in which students attend the courses, as well as recruitment and employability policies, should be carefully considered when examining motivations for enrolment and dropout. Finally, most of the research on this topic was conducted before the COVID-19 pandemic, which changed the working conditions of healthcare systems and may have influenced perceptions among current students who consider nursing a career path [12,13]. In this connection, studies conducted in Italy to describe motivations for enrolment were dated [14,15], with only a study focused on first-year students [14] while two qualitative studies were recently published but not limited to first-year students [16,17]. Internationally, studies that have examined these concepts in the last five years have adopted a considerable variability in outcomes, labeled as dropout [18,37], retention [19,20,21], academic success [22,23], and intention to leave [24,25,26,27,28,29,38], using different approaches. Motivations for enrolment were studied mainly using cross-sectional designs [11,29,30], while motivations for dropout through qualitative methods [16,17,18,36,37]. The exploration of the two phenomena in the same study was carried out only using qualitative approaches, although different concepts (retention and difficulties perceived by students) were investigated in two cases [32,33,34]. In the last ten years, a single study has longitudinally examined the dropout in the BSc Nursing programmes [35], but without providing a link between motivations for enrolment and dropout.

To bridge this knowledge gap, a multicenter longitudinal study has been designed and is now being conducted to evaluate the relationship between motivations for enrolment and dropout in BSc Nursing programmes in Italy. The pilot study presented here was intended as a preliminary investigation that was functional in testing the feasibility of the instruments and refining the procedures that are applied in the multicenter longitudinal study, providing an initial understanding of the link between motivations for enrolment and dropout in first-year nursing students. Given the lack of studies combining different research methods in this field, this pilot study was designed with the purpose of clarifying quantitative analysis of enrolment motivations and providing a deeper interpretation of qualitative findings on dropout motivations, explaining their connection [39].

The aim of this study was to preliminary understand the relationship between motivations for enrolment and the motivations for dropout in first-year undergraduate nursing students.

## 2. Materials and Methods

### 2.1. Study Design

This pilot study applied a multimethod design. This approach consists of two different designs, applied in the same study, used independently (parallely or consequently), but not specifically focused on the same research aim [40]. In this approach, integration is not always required, as this is more frequently pertaining to mixed-methods research traditions. However, in this pilot study, qualitative data are used to provide a subsequent interpretation and clarification of the findings obtained in the quantitative data analysis, as these were gathered from the same population.

Specifically, the two phases focused on two different aspects of the phenomenon of interest (dropout) to understand the relationship between the motivations for enrolment and the motivations for dropout. A first quantitative phase was designed to identify the motivations for enrolment in the BSc Nursing programme; subsequently, a qualitative phase was conducted to explore the motivations for dropout among those participants who discontinued the course.

### 2.2. Setting and Participants

Five universities providing three-year BSc Nursing programmes in the north (S1 and S2), center (S3), and south (S4 and S5) regions of Italy participated in the pilot study from March to September 2023.

In Italy, undergraduate nursing education is composed of theoretical learning (i.e., classroom learning), self-learning activities, clinical placement, and laboratory activities such as simulated learning experiences. University sites generally have dedicated staff who support nursing students during lectures and teaching activities (campus tutors) and nurses who support clinical placement activities (clinical tutors). In each university participant, a contact person and one or more collaborators were identified among campus tutors for students’ recruitment and data collection.

Nursing students were invited to participate if they were enrolled in the first year of the BSc programme for the academic year 2022–2023 at the beginning of the pilot study, before clinical placement. Those who gave their informed consent were included. The nursing education staff identified students who suspended class or clinical placement attendance and discontinued the course using the Student Management Informatic System. Then they were contacted by telephone or email to ask if they were interested in participating in the pilot study.

### 2.3. Ethical Consideration

The study was approved by the Ethics Committee of the University of Torino (protocol no. 0183755 of 15/03/2023—[UOR: SI000045-Classif.III/14]). Participants received verbal and written information about the study. Participation was voluntary and students agreed to complete the questionnaire, specifying their willingness to be contacted for further information. Additional informed consent was obtained from those participating in the interviews. Participants could withdraw from the study at any time and for any reason without consequences. Data were anonymised during transcription, analysis, and presentation by assigning an alphanumerical code to participants, and the results were presented as aggregated. In addition, in accordance with General Data Protection Regulation (EU) 2016/679 [41], protocols for information storage, responsibilities, custody, and data protection were established to ensure participant anonymity.

### 2.4. Data Collection

#### 2.4.1. Quantitative Data/Characteristics of Students and Motivations for Enrolment

The first quantitative phase of this pilot study focused on describing the reasons for enrolling in the BSc Nursing programme. Participants in the study completed a baseline online questionnaire that investigated their characteristics and motivation for enrolment. The questionnaire administered was based on the ‘Motivation Nursing Students Scale’, which was already validated in Italian nursing students [10], with additional reasons for enrolment added from the international literature and the experience of the researchers.

The online questionnaire was designed using LimeSurvey software Version 3.27.22, and comprised 21 questions: 13 closed-ended, four open-ended, and four requiring a preference. Data collected included socio-demographic characteristics of students (gender, age, language, nationality, and educational background), university details, enrolment profile (full-time/part-time), priority selection in admission tests to health professions and other degree programmes, previous university experience, contextual factors (regional origin, distance from home to university, family/work commitments, and financial challenges), source of information, and finally, motivations for enrolment. Students had to indicate their agreement with the statements on a 5-point Likert scale (1 = strongly disagree; 5 = strongly agree) for each of the 27 motivations listed. Motivations included personal experiences that ‘inspired me’, positive experiences with the healthcare system, personal experiences of illness, severe illness of a relative or significant person, past or actual work in the health sector, positive volunteer experiences, having family members in the healthcare sector (nurse or nurse assistant), advice from friends and/or relatives, curriculum counseling sessions, social image of nursing, influence of the COVID-19 pandemic, desire to help those who suffer, feel personally useful, to work closely with people, a long-standing interest in the profession, interest in health-related topics, seeking experience in the healthcare sector, possibility to find a good job immediately after graduation, ambition for career advancement, attraction to a shorter programme or less demanding course than other ones, unsuccessful admission to another university, chance or coincidence, feelings of uncertainty, transitioning from a different faculty due to dissatisfaction, and others.

#### 2.4.2. Qualitative Data/Motivations for Dropout

Participants were followed and tracked until the end of the academic year (in September). Nursing education staff monitored students’ attendance in class and clinical placements to identify potential dropouts and track their enrolment status through the Student Management Informatic System. Additionally, they directly collect relevant communications from students. Among the students who expressed their consent to participate in the qualitative phase, those who had discontinued the course were interviewed to explore their motivations for dropping out.

To achieve credibility and dependability, two research assistants, one PhD student, and one Master of Science in Nursing student trained in qualitative methods with no relationship to participants conducted semi-structured interviews from June 2023 to September 2023 to explore motivations for dropout. Each interview was carried out at a distance, choosing between phone or videocall platform (Webex), and scheduled depending on the students’ preferred time. The interview guide was developed according to the available literature on the phenomenon [11,17,36,42,43] and approved by nursing educators. It comprised five questions: (1) When did you decide to dropout of the course after enrolling?; (2) What motivations led you to drop it? (3) Was one motivation more important than the others? And why?; (4) Did you face any difficulties during your learning experience? (5) Would you like to add a comment on the issues we addressed?

All interviews were audio-recorded. The mean duration of the interviews was 12 min (range 6–17). The recordings were transcribed verbatim, and a researcher checked the accuracy of the transcriptions. Member checking was ensured by asking the participants to validate the transcriptions of their interviews. Due to the specific characteristics of this pilot study, a definition of an adequate sample size to allow data saturation was challenging. However, it was previously shown that 9–17 interviews are sufficient to reach the saturation of qualitative data [44].

### 2.5. Data Analysis

#### 2.5.1. Quantitative Data/Characteristics of Students and Motivations for Enrolment

Data were analysed using Jamovi Statistics software (2.3.28 version) [45] after being imported from the electronic platform that was used to collect data.

Descriptive statistics, medians, and ranges were calculated for continuous variables, while frequencies and percentages were calculated for categorical variables. The Shapiro–Wilk test was used to analyse the normality distribution of the data, while differences between university sites were tested using the Kruskal–Wallis and Chi-square tests. The level of significance was set at *p* < 0.05.

#### 2.5.2. Qualitative Data/Motivations for Dropout

The data were analysed and interpreted following a five-phase process:

1. The transcripts were analysed using an inductive content analysis: they were read several times to acquire a sense of the whole. To be intended as a code, each labelled code must be reported at least three times. The generated codes were then grouped into categories based on their similarities and differences. To ensure trustworthiness and authenticity, two research assistants independently developed the codes and categories and then compared them, and any differences were resolved through discussion. Two researchers not directly involved in the coding and categorisation processes independently validated the analysis.
2. Categorisation was analysed by adopting a deductive approach referring to the Urwin framework [11], at first independently by the two research assistants, and then collegially discussed with the other researchers.
3. Data not fitting the framework formed new supplementary categories.
4. Finally, the motivations for dropout and the relevant difficulties reported by each participant were visually summarised using joint displays to identify emerging patterns among the sample.
5. The findings obtained were functional to corroborate the information collected during the qualitative phase with those reported in the quantitative phase, performed in the discussion.

The ATLAS.ti 8 software [46] was used for efficient data management and coding processes. Significant interview excerpts were labelled using an alphanumeric code (chronological order, university sites) and anonymously analysed.

## 3. Results

### 3.1. Characteristics of Students and Motivations for Enrolment

Of the 973 first-year BSc Nursing students, 423 were from S1, 186 from S2, 184 from S3, 95 from S4 and 85 from S5. Among them, 759 students completed the survey (Table 1): 327 from S1, 149 from S2, 125 from S3, 82 from S4 and 76 from S5. Most were female and Italian, with a median age of 20, mainly from the region in which the universities were located. More than two-thirds (68.3%) graduated from high school, while a quarter had prior university experience, mostly in science and technology (51.2%), although only a fraction had completed it (16.3%). More than half had to travel over 30 min to the university. One-fourth of the sample was composed of active workers, with 6.6% (50 participants) working over 20 h weekly, and nearly 10% (74 participants) had family commitments (e.g., children, elders, etc.). Nursing was the most sought-after health profession among students, with 64.7% applying, followed by midwifery at 13.2% and physiotherapy at 8.7%.

The primary motivations for enrolment in the BSc Nursing programme were the desire to be useful (88.8%) and assist those who suffer (84.3%); also, the opportunity to find employment (74.2%) was relevant. Just above half (52.3%) joined due to personal experience with a loved one’s or family member’s illness, while an equal percentage were attracted by future job prospects (52.3%). Approximately a third of students expressed less motivation to pursue nursing: 22.3% mentioned choosing nursing because of unsuccessful admission to another university, some by chance (5.8%) or by indecision (4.9%); only 5% because they considered it less demanding than other degree courses (Table 2). Some motivations showed significant differences between universities (*p* < 0.05): to gain experience in the care sector, to be in direct contact with people, chance to make a career, positive experience with the health system, already worked or work in the health sector, what I have always wanted to do, COVID-19 Pandemic, social image of nursing, failed to gain admission to another university, and curriculum counselling sessions.

Among these 759 students, 336 (44.3%) declined further contact: 148 from S1, 46 from S2, 93 from S3, 38 from S4 and 11 from S5.

### 3.2. Qualitative Phase/Motivations for Dropout

During the academic year 2022–2023, 141 students (15%) discontinued their attendance to the BSc Nursing programme at the five university sites: 82 from S1, 17 from S2, 27 from S3, 22 from S4, and 3 from S5.

Of them, 54 (38.3%) formally dropped out, 52 (36.9%) communicated their decision to the nurse educators, while 35 (24.8%) no longer showed up for class or placements without giving any notice. Among them, 29 (20.6%) declined further contact, 60 (42.5%) were unable to be reached, and 21 (14.9%) declined the interview. A total of 31 students (26 female and 5 male) were interviewed: 18 (58%) from S1, 7 (22.6%) from S4, 3 (9.7%) from S2, and 3 (9.7%) from S3.

**Figure 1:**
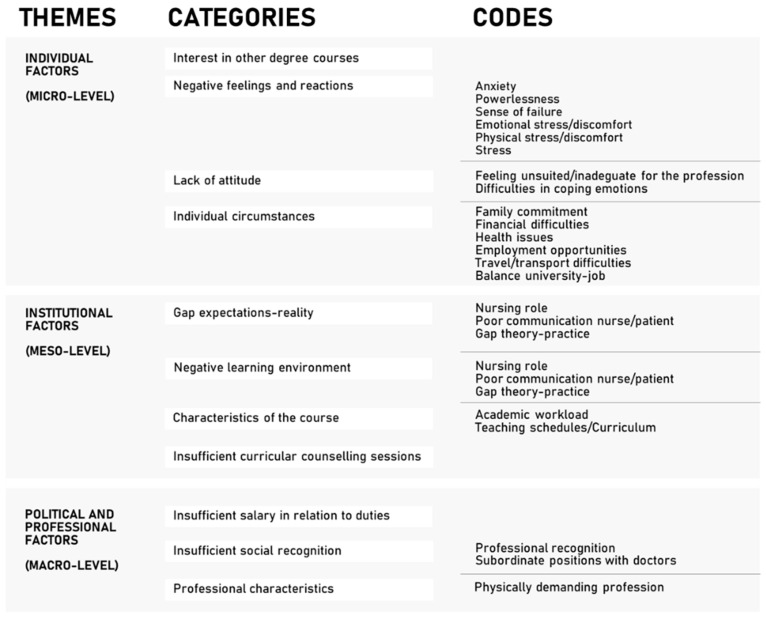
Themes, categories, and codes of interviews.

Eleven categories and three themes were identified as individual (micro-level), institutional (meso-level), political, and professional (macro-level) factors (Figure 1). As each code was reported by participants at least three times to be labelled, though it was not always perceived as a significant difficulty or a reason to discontinue the course.

#### 3.2.1. Individual Factors (Micro Level)

Some students dropped out because they realised they lacked interest in the course and wanted to pursue different curricula that they felt were more suitable for them. These students often hoped to access these alternative courses through the slippage of the ranking order or in the following year. In particular, they do not feel inclined to become nurses:

*“I did not feel like continuing to practice a profession that doesn’t feel suited to me, because I think it deserves the right dedication and passion.”*

(interview 18—S3)

For many students, their first clinical placement was crucial in understanding their professional inclination. Some faced overwhelming challenges in dealing with the clinical environment or caring for sick or elderly people. Even performing certain tasks or activities that require intimate contact with patients, such as venipuncture or personal hygiene, also contributed to dropout:

*“Providing personal hygiene to patients is challenging, particularly for those who are bedridden and require intensive care. The close physical contact involved was emotionally overwhelming.”*

(interview 31—S1)

Other individual factors for nursing students included emotional difficulties. Among them, participants reported anxiety about exam failure and feelings of helplessness in caring for patients, which contributed to dropping out of studies. The difficulty of managing their emotions and gaining awareness of professional responsibilities also played a crucial role for the participants.

*“My anxiety hinders my progress during studies… If I couldn’t pass exams on the first attempt, it would get discouraged.”*

(interview 16—S3)

*“Sometimes, I felt discomfort and uncertainty about the right thing to do, despite it being my first clinical placement and having limited responsibilities, I often felt unease and unable to do something independently.”*

(interview 25—S1)

Also, the personal circumstances related to subsistence influenced some students’ decision to leave the BSc Nursing programme. Among these, the commitment to family and economic challenges related to the need to support themselves or their families were the most commonly reported. However, some participants identified their health condition as the trigger that led them to dropout.

*“I have been taking medication for a few years, which often makes me fatigued... I believe that I cannot cope with academic and professional demands due to this fatigue.”*

(interview 8—S1)

Student workers struggled to balance their university tasks with their jobs, reporting that they often felt vulnerable and unprotected by the system. Commuting and transportation also represented a challenge for some participants, as well as the distance between home and university:

*“It was crucial to join in time the bus stop; missing the bus by just a few minutes meant waiting for the next one and enduring hours of additional travel time. … I would leave home at 06:30 a.m. and return by 06:30 p.m.”*

(interview 5—S1)

#### 3.2.2. Institutional Factors (Meso Level)

The characteristics of the course influenced the dropout due to the excessive workload and teaching schedule. Some students admit that they partially underestimated the course in terms of study load, required activities, and overall commitment needed to complete it:

*“The course is quite dense as it is packed with numerous lectures and internships, many hours… More than anything else, the commitment is a lot.”*

(interview 19—S2)

Transitioning from high school to university required the acquisition of new skills to cope with academic rigour. Several students struggled with the course organisation, such as the division of the courses into multiple modules and interacting with various instructors:

*“I honestly did not appreciate the division into multiple modules; i.e., a single exam split into diverse subjects made it challenging.”*

(interview 3—S1)

Furthermore, participants highlighted the time restrictions due to mandatory attendance and so many hours in class as factors that hinder exam preparation. Students faced similar problems in courses where theory and clinical placement coexist:

*“A significant motivation was the long class schedule. Starting at 8:40 am and ending at 6:00 pm, with optional weekend activities, left little time for studying. By the January exam period, I felt unprepared.”*

(interview 1—S1)

*“Balancing exams, classes, and clinical placement throughout the day was extremely difficult, so I was very tired. A typical day involved clinical placement from 7:00 am to 2:00 pm, then classes from 3:00 pm, exams, and finally another class until 7:00 pm.”*

(interview 13—S4)

The clinical placement allowed students to experience the nursing profession in the field, unveiling the gap between their ideals or expectations, nurtured by the theory class and the working reality. In the real world, some students encountered roles different from what was taught, both in practical and relational terms:

*“Let’s say, while professors described specific nurse roles, the clinical placement revealed a blurring of responsibilities, including tasks like making beds and other duties [...], beyond the expected roles.”*

(interview 10—S4)

*“I regret the lack of interaction with patients during practice. I don’t know if it was only the ward where I was, but the time spent with patients, often just enough to perform a technique, left me sincerely annoyed. Maybe the patient is talking to you and you have to leave. Communication was minimal and I craved more meaningful dialogue and engagement.”*

(interview 6—S1)

An unwelcoming clinical placement environment, marked by dissatisfied nurses or strained relationships with tutors or hospital staff, sometimes also marred by the student’s poor preparation, negatively impacted their experience, influencing some dropouts.

*“My strained relationship with the internship tutor hindered open communication, which affected my overall experience. Therefore, I did not feel free to talk to him.”*

(interview 28—S1)

#### 3.2.3. Political and Professional Factors (Macro Level)

An important issue related to labour policies was the limited financial recognition given to the tasks performed by nurses, which the participants had direct experience with during their clinical placements, which impacted their decision to leave:

*“Even from a purely economic perspective, in my opinion, what you do is underpaid.”*

(interview 7—S1)

Other trainees reported disillusionment with the profession, citing a negative image, low social prestige, and subordinate positions with doctors in universities and clinics. The importance and autonomy of their role tend to be underestimated, even by the students themselves, who often change their minds only after completing their clinical placements and having experienced the complexity of the professional role:

*“I expected a greater appreciation of a role that I believe is truly fundamental. During the clinical placement, I witnessed several scenes in which nurses or we trainees were dismissed, as well as some not nursing professors who emphasized the limited competence of nurses compared to doctors.”*

(interview 18—S3)

Lastly, the physical exertion required to work in certain environments was not only difficult but also a significant factor cited as a motivation for dropout:

*“Yes, objectively, it’s a physically demanding profession, so even during training shifts, it can be exhausting, requiring you to stand for up to 8 h.”*

(interview 8—S1)

A mind map clarifying the relationships between categories and the phenomenon of nursing student dropout is available in the Supplementary Materials (Figure S1).

#### 3.2.4. Quantitative Content Analysis

**Figure 2:**
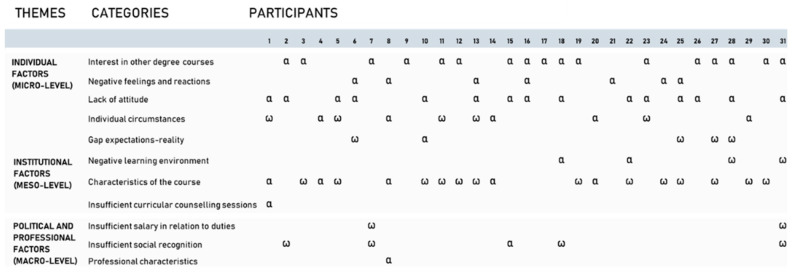
Students’ relevant difficulties and motivations for dropout. α motivations for dropout; ω relevant difficulties.

Of the 31 students interviewed (Figure 2), more than half (17 students) dropped out of the BSc Nursing programme because they were interested in other degree courses; for most of them (15 students), this decision was associated with other motivations for dropout. Among the decisions associated, there were the lack of aptitude (7 students), negative feelings or reactions (1 student), and insufficient social recognition (1 student). Six of the fifteen students who dropped out because they were interested in other degree courses reported the characteristics of the course as a relevant challenge. Additionally, nearly half (13 students) reported that the characteristics of the course were a significant difficulty. Only five students dropped out due to personal circumstances such as financial difficulties, family, health problems, or the impossibility of balancing university and work commitments. These last conditions are always combined with the characteristics of the course. Political and professional factors are less frequent and rarely influence dropping out.

## 4. Discussion

This pilot study applied a multimethod approach to initiate a comprehensive understanding of the connection existing between the motivations for enrolment and dropout of first-year students attending the BSc Nursing programmes.

The strongest motivations reported by students to enter the nursing profession are the desire to be useful and to help those who suffer. Despite social and cultural changes, gaining a sense of fulfilment through helping people still characterises the aid professions [47], especially nursing [8]. The nature of the relationship of caregiving, intrinsic in the nursing role, allows direct contact with people [48], a factor identified by students as one of the main reasons for enrolment. Furthermore, the influence of personal or loved ones’ experiences of illness on the individual desire to help others has already been identified [49]. Those who enrolled in nursing had a more vivid recollection of such experiences than those who chose non-health-related university courses, and it is likely that seeing nurses’ work first-hand inspired a desire to pursue the same career [15]. Our study corroborates this statement since more than half of the students reported that direct and indirect illness experiences were their motivation to join the nursing career.

In contemporary society, the media plays a crucial role in shaping public perceptions of nurses. Media exposure can influence the willingness of students to become nurses [49], even if these instruments are used to show a distorted representation of the profession, which is based mainly on misconceptions and stereotypes that lead to a gap between public perception and actual professional responsibility [50], which can have a negative impact on the image of nursing. The latter could vary between countries and cultural contexts, but a more positive image of nursing has been shown to enhance the likelihood that students would choose the profession [51]. Regarding the mediatic impact, the importance of the nursing role was strongly highlighted during the recent COVID-19 pandemic, emphasising that nurses are on the front lines of the healthcare system and on the patient side, where they face daily challenges to provide quality patient care, sometimes with limited resources [52]. Despite this, only one-third of our participants considered the image of nurses and the pandemic as significant factors in their decision to enrol in the BSc Nursing programmes.

Another primary motivation reported by students was the possibility of finding a job that provides independence and economic security compared to their peers. In Italy, nursing is one of the professions with the highest employment rate one year after graduation [53]. Economic stability is generally accompanied by career advancement opportunities, which are limited for nurses. This aspect may underlie the interest of new generations in various areas of professional development, including healthcare [54].

The integration of quantitative and qualitative findings resulted in various reflection points. Firstly, students who are less motivated to pursue nursing as a secondary option could more likely dropout than their peers, as previously highlighted [55]. Around a third of the participants opt for nursing as a secondary choice, often due to exclusion from their preferred degrees, uncertainty, or coincidence. In countries with limited enrolment measures, nursing has become attractive due to the abundance of places available compared to programmes such as medicine, physiotherapy, or midwifery, and the option to validate exams in case of shifts from one course to another [8]. Our findings show that a primary interest in other degrees could represent a relevant motivation for first-year dropouts among participants. On the other hand, as previously reported, students who prioritise nursing show a stronger professional identity and are more inclined to continue in this course [56]. A positive professional attitude can foster the formation of a professional identity [57]; however, this attribute seems lacking in our students who consider nursing a secondary choice. Almost all of them used expressions such as “it was not my path” or “it wasn’t meant for me”, reflecting the descriptions that were already provided as reasons for dropout in an Italian context [17].

Secondly, students who underestimate the demands of a nursing degree may face strong difficulties related to the characteristics of the course that could lead to dropout. In fact, participants frequently cited excessive academic workload and tight teaching schedules as institutional stressors. Some students interviewed acknowledged that they underestimated the required workload and time demands in classes, which affected their social life [58,59]. This factor could have been particularly burdensome for the few participants who had to leave due to individual circumstances, such as family or work commitments, health problems, or transport. Previous qualitative research [16,17,60] highlighted that handling various responsibilities related to family, education, employment, or commuting long distances between home and university led to difficulties in time management, especially among older students, resulting in dropouts. In particular, in our study, 25% of students held jobs, requiring a balance between education and work. This combination of individual factors may represent a relevant trigger for dropout.

Our findings also highlight the potential role of clinical placement in sustaining recognition in the nursing role or in deciding to dropout of the course [61]. In fact, unmotivated students reported that they found confirmation of their lack of attitude during their first clinical experience [62]. Moreover, it has already been highlighted how students, although motivated, sometimes realised that they lack the emotional and physical resources required to become nurses when exposed to the professional reality of the clinical environment and interactions with ill and suffering patients [17]. Conversely, students may experience disillusionment due to a gap between expectations and reality potentially caused by over-idealising the profession [58,59]. They faced limited person-centred care and discrepancies in the nursing role [17,62]. Furthermore, being in an unwelcoming environment with dissatisfied nurses could negatively affect their intention to stay [11]. Experiencing a failing tutorial relationship or encountering negative role models could further discourage students, leading to physical discomfort and negative feelings such as anxiety, uncertainty, inadequacy, or powerlessness [16,60,62].

In this pilot study, motivations for dropout related to political and professional factors, such as insufficient social recognition, limited salary in relation to duties, and professional characteristics like physical demand, seem less prevalent. The public still sees nursing as a low-status profession, subordinate to physicians, not requiring any academic qualifications, and lacking professional autonomy [50], but sometimes students still experience certain subordination dynamics in the clinical environment. These last factors may hinder student retention, but probably also enrolment.

Finally, previous studies highlighted how gender disparity could affect dropout rates, particularly for male students who are an underrepresented population in nursing and may experience negative learning environments characterised by stereotypes, prejudice, and discrimination [63,64]. However, our participants did not report experiencing these adverse conditions. This suggests that, while existing research points to a significant issue, the specific context of the participants may differ from broader trends.

Taking into account the dropout patterns identified among those participants, university institutions should consider what investments could benefit these students. More extensive orientation initiatives in high schools could encourage selective enrolment. In addition, appropriate curricular counselling sessions or targeted interventions during the learning pathway of students for which nursing is not a first choice could improve their engagement in the BSc Nursing programme. Among these strategies, previous studies suggested improving students’ preparation prior to beginning their studies with additional information and advice about time management, prioritisation of academic skills, and clinical requirements [65]. Moreover, it could be useful to establish a peer support environment [66] and develop student engagement approaches such as group work, direct contact, face-to-face sessions, and simulation laboratory sessions to share their knowledge and learning experiences, create collective meaning, develop theoretical understandings, and share experiences from their own backgrounds [67]. Other solutions, such as mindfulness practice, could represent feasible interventions to support the well-being of students [68]. University institutions should support students who juggle multiple personal commitments, particularly among older individuals, where the prospect of a secure job may attract enrolment in the BSc Nursing programmes. Finally, extensive efforts among nurses’ educators and clinical staff should be made to provide a positive and authentic clinical learning environment to promote nursing student satisfaction and their sense of belonging and retention [56,69]. Further studies should focus on predictive analytics of students who show lower motivation and determine how many students end up discontinuing. The effectiveness of interventions aimed at improving the motivation and the sense of belonging of students interested in other courses or for whom nursing is not the first choice should be studied, as such interventions could represent a viable solution to limit the shortage of nurses in the medium term.

The limitations of this pilot study are mainly related to the convenience sampling method applied, which may have affected the sample representativeness. In fact, the sites where data were collected contributed heterogeneously to the sample, which may have been unbalanced due to the presence of students more willing to participate. Moreover, the data collection period, which was widespread in the five sites and opened after the beginning of the lessons, could have limited a full representation of all students who had already discontinued because unreachable. Furthermore, the dropout rate found, the limited willingness to be further contacted by half of the students, and the difficulty of tracking down students willing to be interviewed could have limited the number of potential interviews. Nevertheless, 31 students, representing 22% of those students who discontinued the BSc Nursing programme, were interviewed, allowing adequate data saturation and representativeness [44]. Additionally, although some students agreed to participate and were interviewed, they did not complete the enrolment motivation questionnaire, limiting the full corroboration of data. Finally, the use of semi-structured interviews could have led to interviewer bias, as they could have influenced the responses given by interviewees. Nevertheless, the presence of an adequate number of researchers involved in the data collection and analysis processes and their external position ensured trustworthiness and data triangulation. Moreover, the use of a validated tool to collect data on motivations for enrolment, customising this with a series of contextual information that was applied in five universities, allowed us to portray timely the situation in the Italian BSc Nursing student population.

## 5. Conclusions

Although intended as a preliminary investigation, this pilot study could allow an initial understanding of the complex relationship between motivations for enrolment and dropout of first-year nursing students, providing valuable information for future studies and efforts by policymakers to address the nursing shortage. The findings obtained should promote the development of personalised interventions by faculties to support those students who are less motivated to enrol, as they could be the most likely to dropout. Additionally, the role of clinical placements in dropping out of the course should be studied and addressed, as it is identified as a pivotal moment in which students face their future professional roles. Future longitudinal studies, such as the multicenter cohort study that is currently ongoing in Italy and designed starting from this inquiry, are needed to deepen the understanding of the phenomena.

## Figures and Tables

**Table 1:** Socio-demographic characteristics of participants (N = 759).

	N	%
**Age** Median (range)	20	(18–51)
**Gender** Female	619	81.6
**Nationality** Italian	701	92.2
**Secondary School**		
High School	518	68.3
Professional or technical education	241	31.7
Prior university experience *	203	26.6
Graduated	33	15.8
Area *: Scientific-technological		
	104	51.2
Humanistic-social	50	24.6
Health	44	21.7
Others	4	2.0
**Origin from university**		
Same region	617	81.3
Other region	136	17.9
Foreign country	6	0.8
**Distance home-university** ≥30 min	478	63.0
**Family commitments**	74	9.7
**Work commitments**	190	25.1
1–10 h/week	87	11.5
11–20 h/week	53	7.0
More than 20 h/week	50	6.6
**Full time**	750	98.8

* Missing.

**Table 2:** Motivations for enrolment.

Motivations	N	%
With this job I can make myself useful	674	88.8
Because it is a profession that will allow me to help those who suffer	640	84.3
I am interested in health subjects	628	82.7
I want to gain experience in the care sector	622	81.9
Because it is a profession that will allow me to be in direct contact with people	576	75.9
This degree course will give me the opportunity to find a good job immediately after graduation	563	74.2
Because there is a chance to make a career	397	52.3
Illness experience of family members or loved ones	397	52.3
A personal experience ‘inspired me’	351	46.2
I have family members working in the health sector	344	45.3
I had positive experience with the health system	332	43.7
I have family members or contacts nurses or nurse assistants	320	42.2
That is what I have always wanted to do	273	36.0
COVID-19 Pandemic	231	30.4
Social image of nursing	227	29.9
I have had positive experiences in volunteering	204	26.9
It is a degree course shorter than other ones	180	23.7
I was advised by friends and/or relatives	177	23.3
I failed to gain admission to another university	169	22.3
Personal illness experience	151	19.9
It is a degree course with more availability than other ones	148	19.5
I have already worked or work in the health sector	105	13.8
For curriculum counselling sessions	104	13.7
I come from another faculty where I was not happy/motivated	81	10.7
By chance or by coincidence	44	5.8
It is a degree course less demanding than the other ones	39	5.1
I did not know what to do	37	4.9
Others	5	0.7

## Data Availability

The data and materials supporting the findings of this study are available from the corresponding author upon reasonable request.

## Supplementary Materials

The following supporting information can be downloaded at: https://www.mdpi.com/article/10.3390/nursrep14040254/s1, Figure S1: Mind map: motivations for dropout.

## Public Involvement Statement

No public involvement in any aspect of this research.

## Guidelines and Standards Statement

This manuscript was drafted against the Strengthening the Reporting of Observational Studies in Epidemiology (STROBE) Statement guidelines for reporting observational research.

## References

[1] Pressley C., Garside J. (2023). Safeguarding the Retention of Nurses: A Systematic Review on Determinants of Nurse’s Intentions to Stay. Nurs. Open.

[2] Bucham J., Catton H. (2023). Recover to Rebuild: Investing in the Nursing Workforce for Health System Effectiveness.

[3] World Health Organization (2020). Global Strategy on Human Resources for Health: Workforce 2030.

[4] World Health Organization (2021). The WHO Global Strategic Directions for Nursing and Midwifery (2021–2025).

[5] OECD (2023). Health at a Glance 2023: OECD Indicators.

[6] Federazione Nazionale degli Ordini delle Professioni Infermieristiche, D.B. Incontro FNOPI-CRUI: La buona università può fare la differenza. *Fnopi* 2024. https://www.fnopi.it/2024/07/04/fnopi-crui/.

[7] Ministero dell’Università e della Ricerca Decreto Ministeriale n. 1119 Del 01-08-2024|Ministero Dell’Università e Della Ricerca. https://www.mur.gov.it/it/atti-e-normativa/decreto-ministeriale-n-1119-del-01-08-2024.

[8] Mastrillo A., Bevilacqua L., Cenerelli E. (2023). Report: Corsi di Laurea delle Professioni Sanitarie. Dati sull’accesso ai corsi e Programmazione posti nell’ A.A. https://presidenti-medicina.it/corsi-di-laurea-delle-professioni-sanitarie-dati-sullaccesso-ai-corsi-e-programmazione-dei-posti-nell-a-a-2023-24/.

[9] Raymond A., James A., Jacob E., Lyons J. (2018). Influence of Perceptions and Stereotypes of the Nursing Role on Career Choice in Secondary Students: A Regional Perspective. Nurse Educ. Today.

[10] Bulfone G., Badolamenti S., Biagioli V., Maurici M., Macale L., Sili A., Vellone E., Alvaro R. (2021). Development and Psychometric Evaluation of the Motivation for Nursing Student Scale (MNSS): A Cross Sectional Validation Study. Int. J. Nurs. Educ. Scholarsh..

[11] Urwin S., Stanley R., Jones M., Gallagher A., Wainwright P., Perkins A. (2010). Understanding Student Nurse Attrition: Learning from the Literature. Nurse Educ. Today.

[12] Avraham R., Wacht O., Yaffe E., Grinstein-Cohen O. (2023). Choosing a Nursing Career during a Global Health Event: A Repeated Cross-Sectional Study. Nurse Educ..

[13] Yavaş G., Özerli A.N. (2023). The Public Image of Nursing during the COVID-19 Pandemic: A Cross-Sectional Study. Int. Nurs. Rev..

[14] Dante A., Graceffa G., Del Bello M., Rizzi L., Ianderca B., Battistella N., Bulfone T., Grando R., Zuliani S., Casetta A. (2014). Factors Influencing the Choice of a Nursing or a Non-Nursing Degree: A Multicenter, Cross-Sectional Study. Nurs. Health Sci..

[15] Di Giulio P., Domenighetti G., Tomada A., Baseotto S. (2016). Contact with death or illness and career choice in non-medical health professions and business students: A cross-sectional analysis. Assist. Inferm. Ric..

[16] Mazzotta R., Durante A., Bressan V., Cuoco A., Vellone E., Alvaro R., Bulfone G. (2024). Perceptions of Nursing Staff and Students Regarding Attrition: A Qualitative Study. Int. J. Nurs. Educ. Scholarsh..

[17] Canzan F., Saiani L., Mezzalira E., Allegrini E., Caliaro A., Ambrosi E. (2022). Why Do Nursing Students Leave Bachelor Program? Findings from a Qualitative Descriptive Study. BMC Nurs..

[18] Soerensen J., Nielsen D.S., Pihl G.T. (2023). It’s a Hard Process—Nursing Students’ Lived Experiences Leading to Dropping out of Their Education; a Qualitative Study. Nurse Educ. Today.

[19] Dries K.J. (2020). Variables Impacting Program Completion of Readmitted Associate Degree Nursing Students. Nurse Educ..

[20] Simpson O., Bennett C.L., Whitcombe S.W. (2024). Student Nurse Retention. Lived Experience of Mature Female Students on a UK Bachelor of Nursing (Adult) Programme: An Interpretative Phenomenological Analysis. J. Adv. Nurs..

[21] Liu X.-L., Nic Giolla Easpaig B., Garti I., Bressington D., Wang T., Wikander L., Tan J.-Y.B. (2024). Improving Success and Retention of Undergraduate Nursing Students from Rural and Remote Australia: A Multimethod Study Protocol. Nurse Educ. Pract..

[22] Bulfone G., Iovino P., Mazzotta R., Sebastian M., Macale L., Sili A., Vellone E., Alvaro R. (2022). Self-Efficacy, Burnout and Academic Success in Nursing Students: A Counterfactual Mediation Analysis. J. Adv. Nurs..

[23] Bulfone G., Mazzotta R., Cocco M., Maurici M., Anastasia M., Macale L., Sili A., Vellone E., Alvaro R. (2022). Variables Predicting Academic Success of Nursing Students: A Longitudinal Study in a Nursing Bachelor’s Degree Program. Ann. Ig..

[24] Hong Q.N., Pluye P., Bujold M., Wassef M. (2017). Convergent and Sequential Synthesis Designs: Implications for Conducting and Reporting Systematic Reviews of Qualitative and Quantitative Evidence. Syst. Rev..

[25] Matteau L., Toupin I., Ouellet N., Beaulieu M., Truchon M., Gilbert-Ouimet M. (2023). Nursing Students’ Academic Conditions, Psychological Distress, and Intention to Leave School: A Cross-Sectional Study. Nurse Educ. Today.

[26] Avilés-González C.I., Curcio F., Dal Molin A., Casalino M., Finco G., Galletta M. (2024). Relationship between Tutor Support, Caring Self-Efficacy and Intention to Leave of Nursing Students: The Roles of Self-Compassion as Mediator and Moderator. Int. J. Nurs. Educ. Scholarsh..

[27] Kandil F., El Seesy N., Banakhar M. (2021). Factors Affecting Students’ Preference for Nursing Education and Their Intent to Leave: A Cross-Sectional Study. Open Nurs. J..

[28] Martinez M.C., de Oliveira M.D.R.D., Fischer F.M. (2022). Factors Associated with Work Ability and Intention to Leave Nursing Profession: A Nested Case-Control Study. Ind. Health.

[29] Kox J.H.A.M., Runhaar J., Groenewoud J.H., Bierma-Zeinstra S.M.A., Bakker E.J.M., Miedema H.S., Roelofs P.D.D.M. (2022). Do Physical Work Factors and Musculoskeletal Complaints Contribute to the Intention to Leave or Actual Dropout in Student Nurses? A Prospective Cohort Study. J. Prof. Nurs..

[30] Ait Ali D., Ncila O., Ouhhamou S., Rizzo A., Chirico F., Khabbache H. (2024). Motivations Driving Career Choices: Insights From a Study Among Nursing Students. SAGE Open Nurs..

[31] Jiang H., Mei Y., Wang X., Zhao Z., Lin B., Wang W., Zhang Z. (2023). Professional Calling among Nursing Students: A Latent Profile Analysis. BMC Nurs..

[32] Ten Hoeve Y., Castelein S., Jansen W.S., Jansen G.J., Roodbol P.F. (2017). Nursing Students’ Changing Orientation and Attitudes towards Nursing during Education: A Two Year Longitudinal Study. Nurse Educ. Today.

[33] Teresa-Morales C., Rodríguez-Pérez M., Ramos-Pichardo J.D. (2023). Reasons for Choosing and Completing Nursing Studies among Incoming and Outgoing Students: A Qualitative Study. Nurse Educ. Today.

[34] Wynne S., Garrow A. (2024). Exploring the Motivations, Expectations, and Experience of Graduate-Entry Nursing Students: A Qualitative Research Study. Nurse Educ. Today.

[35] Torregosa M.B., Patricio O. (2024). Predictors of Attrition and Program Dismissal in a Nursing Major. Nurse Educ. Today.

[36] Kukkonen P., Suhonen R., Salminen L. (2016). Discontinued Students in Nursing Education—Who and Why?. Nurse Educ. Pract..

[37] Dancot J., Pétré B., Dardenne N., Donneau A.-F., Detroz P., Guillaume M. (2021). Exploring the Relationship between First-Year Nursing Student Self-Esteem and Dropout: A Cohort Study. J. Adv. Nurs..

[38] Arianta K., Goller M. (2024). Dynamics of Persistence, Withdrawal, and Dropout Intentions in the Initial Phase of Nursing Training: A Qualitative Longitudinal Study. Empir. Res. Vocat. Educ. Train..

[39] Shorten A., Smith J. (2017). Mixed Methods Research: Expanding the Evidence Base. Evid.-Based Nurs..

[40] Anguera M.T., Villaseñor A., Losada J., Sanchez-Algarra P., Onwuegbuzie A. (2018). Revisiting the Difference between Mixed Methods and Multimethods: Is It All in the Name?. Qual. Quant..

[41] Regulation (EU) 2016/679 of the European Parliament and of the Council of 27 April 2016 on the Protection of Natural Persons with Regard to the Processing of Personal Data and on the Free Movement of Such Data, and Re-pealing Directive 95/46/EC (General Data Protection Regulation) (Text with EEA Relevance) (OJ L 119 04.05.2016, p. 1). https://eur-lex.europa.eu/eli/reg/2016/679/oj).

[42] Jeffreys M.R. (2022). Nursing Student Retention and Success: Action Innovations and Research Matters. Teach. Learn. Nurs..

[43] Glossop C. (2002). Student Nurse Attrition: Use of an Exit-Interview Procedure to Determine Students’ Leaving Reasons. Nurse Educ. Today.

[44] Hennink M., Kaiser B.N. (2022). Sample Sizes for Saturation in Qualitative Research: A Systematic Review of Empirical Tests. Soc. Sci. Med..

[45] Jmv r Package—Jamovi. https://www.jamovi.org/jmv/.

[46] (2021). ATLAS.Ti Scientific Software Development GmbH.

[47] Macdiarmid R., Turner R., Winnington R., McClunie-Trust P., Donaldson A., Shannon K., Merrick E., Jones V., Jarden R. (2021). What Motivates People to Commence a Graduate Entry Nursing Programme: A Mixed Method Scoping Review. BMC Nurs..

[48] Messineo L., Allegra M., Seta L. (2019). Self-Reported Motivation for Choosing Nursing Studies: A Self-Determination Theory Perspective. BMC Med. Educ..

[49] Allen L.M., Cooper S.J., Missen K. (2022). Bachelor of Science in Nursing Students’ Perceptions of Being a Nurse: A Scoping Review. J. Prof. Nurs..

[50] ten Hoeve Y., Jansen G., Roodbol P. (2014). The Nursing Profession: Public Image, Self-Concept and Professional Identity. A Discussion Paper. J. Adv. Nurs..

[51] Ben Natan M., Becker F. (2010). Israelis’ Perceived Motivation for Choosing a Nursing Career. Nurse Educ. Today.

[52] Blau A., Sela Y., Grinberg K. (2023). Public Perceptions and Attitudes on the Image of Nursing in the Wake of COVID-19. Int. J. Environ. Res. Public Health.

[53] AlmaLaurea Laureati Nelle Professioni Sanitarie: Focus Sulle Retribuzioni. https://www.almalaurea.it/i-dati/le-nostre-indagini/indagini-tematiche/laureati-nelle-professioni-sanitarie-focus-sulle-retribuzioni.

[54] Wallis L., Locke R., Ryall S., Harden B. (2023). Motivations for Choosing an Allied Health Profession Career: Findings from a Scoping Review. Int. J. Pract.-Based Learn. Health Soc. Care.

[55] Salamonson Y., Everett B., Cooper M., Lombardo L., Weaver R., Davidson P.M. (2014). Nursing as First Choice Predicts Nursing Program Completion. Nurse Educ. Today.

[56] Al-Noumani H., Al Zaabi O., Arulappan J., George H.R. (2024). Professional Identity and Preparedness for Hospital Practice among Undergraduate Nursing Students: A Cross-Sectional Study. Nurse Educ. Today.

[57] Kurt Y., Turhal E., Batmaz F. (2024). Nursing Students’ Processes of Taking Role Models and Being Role Models: A Descriptive Phenomenological Study. Nurse Educ. Today.

[58] Williamson G.R., Health V., Proctor-Childs T. (2013). Vocation, Friendship and Resilience: A Study Exploring Nursing Student and Staff Views on Retention and Attrition. Open Nurs. J..

[59] O’Donnell H. (2011). Expectations and Voluntary Attrition in Nursing Students. Nurse Educ. Pract..

[60] Andrew S., Salamonson Y., Weaver R., Smith A., O’Reilly R., Taylor C. (2008). Hate the Course or Hate to Go: Semester Differences in First Year Nursing Attrition. Nurse Educ. Today.

[61] Eick S.A., Williamson G.R., Heath V. (2012). A Systematic Review of Placement-Related Attrition in Nurse Education. Int. J. Nurs. Stud..

[62] Bakker E.J.M., Verhaegh K.J., Kox J.H.A.M., van der Beek A.J., Boot C.R.L., Roelofs P.D.D.M., Francke A.L. (2019). Late Dropout from Nursing Education: An Interview Study of Nursing Students’ Experiences and Reasons. Nurse Educ. Pract..

[63] Salamonson Y., Maneze D., Smith B.W., Duff J., Theobald K.A., Montayre J., McTier L., Donnelly F. (2023). Are Men Treated Differently in Clinical Placements during Nursing Studies? A Cross-Sectional Study. J. Clin. Nurs..

[64] Lindenfeld M. (2024). Nursing Students Who Identify as Men; Efficacy and Persistence. Nurse Educ. Pract..

[65] Sheedy A.D. (2024). Qualitative Research into Study Preparation Recommendations to Facilitate Role Adaptation as a Student Nurse. Aust. J. Adv. Nurs..

[66] Cupelli L.M., Colalillo G.C. (2024). Implementing Peer Learning to Enhance Academic Performance in First-Year Nursing Students. Teach. Learn. Nurs..

[67] Yen M., Koo T.F., Sattarshetty K., Doan D., Alsharaydeh E. (2024). International Graduate Entry Nursing Students: A Qualitative Study on Engagement. Nurse Educ. Pract.

[68] Strout K., Schwartz-Mette R., Parsons K., Sapp M. (2024). The Scholarship of Wellness and Mindfulness to Support First-Year Nursing Students’ Response to Stress. Nurs. Educ. Perspect..

[69] Browne C., Wall P., Batt S., Bennett R. (2018). Understanding Perceptions of Nursing Professional Identity in Students Entering an Australian Undergraduate Nursing Degree. Nurse Educ. Pract..

