# Supplementary figures and images for: Motivations for Enrolment and Dropout of First-Year Undergraduate Nursing Students: A Pilot Multimethod Study
Source: Nurs Rep. 2024 Nov 13;14(4):3488–504. doi: 10.3390/nursrep14040254 (PMC11587445; doi:10.3390/nursrep14040254)

**Figure S1.** Mind map: motivations for dropout

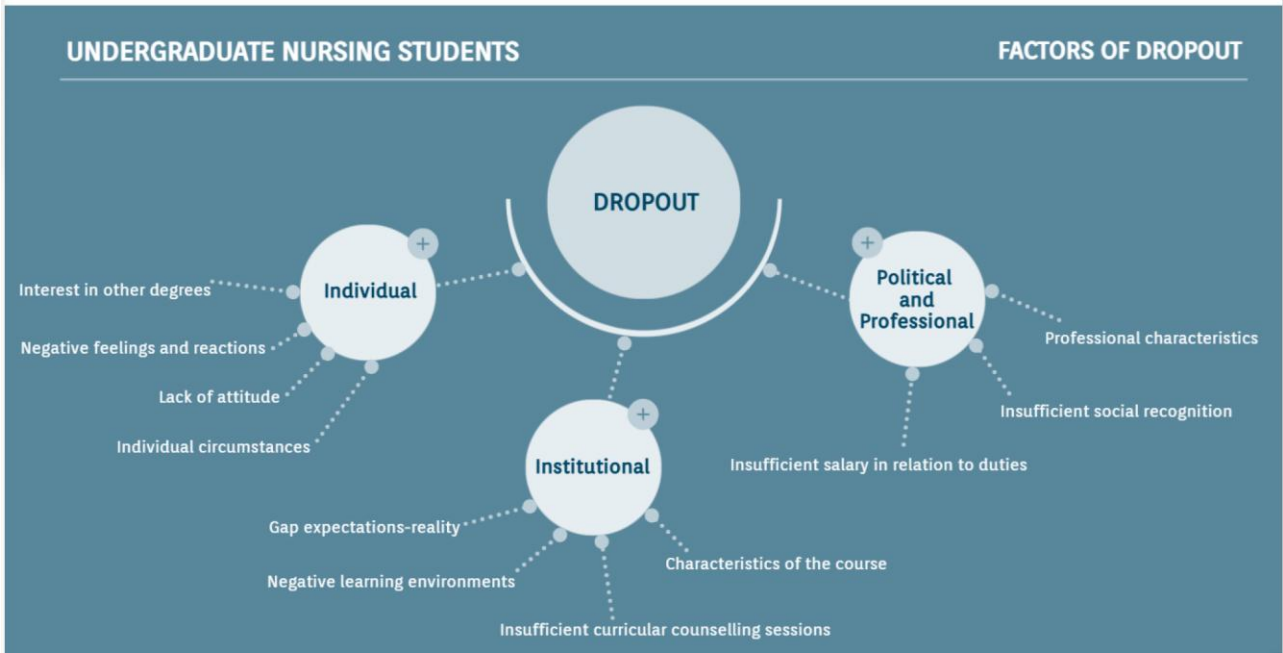

Supplement: Supplementary file 1 [file nursrep-14-00254-s001.zip › nursrep-3278283-supplementary.pdf]
